# Evolutionary Analyses of Base-Pairing Interactions in DNA and RNA Secondary Structures

**DOI:** 10.1093/molbev/msz243

**Published:** 2019-10-30

**Authors:** Michael Golden, Benjamin Murrell, Darren Martin, Oliver G Pybus, Jotun Hein

**Affiliations:** 1 Department of Statistics, University of Oxford, Oxford, United Kingdom; 2 Department of Zoology, University of Oxford, Oxford, United Kingdom; 3 Department of Microbiology, Tumor and Cell Biology, Karolinska Institutet, Stockholm, Sweden; 4 Department of Integrative Biomedical Sciences, Computational Biology Group, Institute of Infectious Disease and Molecular Medicine, University of Cape Town, Cape Town, South Africa

**Keywords:** evolution, nucleic acid structure, coevolution, probabilistic model

## Abstract

Pairs of nucleotides within functional nucleic acid secondary structures often display evidence of coevolution that is consistent with the maintenance of base-pairing. Here, we introduce a sequence evolution model, MESSI (Modeling the Evolution of Secondary Structure Interactions), that infers coevolution associated with base-paired sites in DNA or RNA sequence alignments. MESSI can estimate coevolution while accounting for an unknown secondary structure. MESSI can also use graphics processing unit parallelism to increase computational speed. We used MESSI to infer coevolution associated with GC, AU (AT in DNA), GU (GT in DNA) pairs in noncoding RNA alignments, and in single-stranded RNA and DNA virus alignments. Estimates of GU pair coevolution were found to be higher at base-paired sites in single-stranded RNA viruses and noncoding RNAs than estimates of GT pair coevolution in single-stranded DNA viruses. A potential biophysical explanation is that GT pairs do not stabilize DNA secondary structures to the same extent that GU pairs do in RNA. Additionally, MESSI estimates the degrees of coevolution at individual base-paired sites in an alignment. These estimates were computed for a SHAPE-MaP-determined HIV-1 NL4-3 RNA secondary structure. We found that estimates of coevolution were more strongly correlated with experimentally determined SHAPE-MaP pairing scores than three nonevolutionary measures of base-pairing covariation. To assist researchers in prioritizing substructures with potential functionality, MESSI automatically ranks substructures by degrees of coevolution at base-paired sites within them. Such a ranking was created for an HIV-1 subtype B alignment, revealing an excess of top-ranking substructures that have been previously identified as having structure-related functional importance, among several uncharacterized top-ranking substructures.

## Introduction

The primary role of nucleic acid molecules, such as deoxyribonucleic acid (DNA) and ribonucleic acid (RNA), is to encode genetic information for storage and transfer. However, both types of molecules can form structures with additional functions (Mattick 2003). DNA is ordinarily thought of as a double-stranded molecule forming the now iconic double helical configuration (Watson and Crick 1953), although many viral genomes consist entirely of single-stranded DNA (ssDNA) or single-stranded RNA (ssRNA) molecules. Such single-stranded nucleic acid molecules are far less constrained than double-stranded ones in the variety of functional structures that they can form. For example, the Rev response element (RRE) within the single-stranded HIV RNA genome plays a crucial role in the regulation of HIV replication by binding the HIV Rev protein to facilitate the transfer of HIV genomes from the nucleus to the cytoplasm where translation and virion packaging occur (Heaphy et al. 1990; Daugherty et al. 2010).

The structures that nucleic acid molecules form are commonly referred to as their secondary or tertiary structures. Secondary structure is defined as the set of hydrogen bonding interactions between the constituent bases of a nucleic acid molecule; tertiary structure is defined as the arrangement of the constituent atoms of a nucleic acid molecule in 3D space. This study focuses exclusively on RNA and DNA secondary structures.

Both computational (Bernhart et al. 2006; Markham and Zuker 2008; Sükösd et al. 2012) and hybrid experimental-computational techniques (Wilkinson et al. 2006) for secondary structure prediction exist. However, even if the secondary structure of an RNA sequence can be accurately determined, this does not immediately say anything about the potential functional or biological importance of the identified structure. Many RNA secondary structures are known to have specific biological functions, and it is expected that evolutionary conservation or adaptation of these structures might detectably impact patterns of sequence diversity and evolution.

One evolutionary signal that can be used to identify selectively maintained secondary structures is nucleotide coevolution. Nucleotide coevolution is expected at base-paired nucleotide positions within RNA and DNA secondary structures (Eddy and Durbin 1994; Tuplin et al. 2002; Cheng et al. 2012). Many pairs of nucleotides within RNA molecules exhibit evidence of coevolution, such that whenever a substitution occurs in one partner of the pair, complementary substitutions are selected for in the other partner in a manner that is consistent with the selective maintenance of canonical base-pairing (Cheng et al. 2012). The restricted nature of base-pairing interactions in nucleic acid structures (compared with amino acid interactions in protein structures) permits both nucleic acid structural conformations and nucleotide coevolution to be predicted with relative ease. In this study, we consider the canonical RNA base-pairs to be the two Watson–Crick base-pairs, GC and AU, and the weaker GU wobble base-pair (GC, AT, and GT base-pairs in DNA, respectively).

Methods for detecting coevolution, such as mutual information (Eddy and Durbin 1994; Lindgreen et al. 2006), can be used to aid the computational inference of secondary structures. Accordingly, some RNA comparative secondary structure prediction approaches, such as PPfold (Sükösd et al. 2012), use information about coevolving nucleotides inferred from sequence alignments to more accurately predict secondary structures. Conversely, within a given secondary structural element, evidence that paired bases are coevolving is evidence of the functional importance of that element (Tuplin et al. 2002; Cheng et al. 2012; Muhire et al. 2014).

Standard approaches for measuring coevolution (or more accurately: covariation), such as mutual information, are nonevolutionary in that they do not take into account the phylogenetic relationships of the sequences being analyzed. Founder substitutions can, by chance, induce correlations between bases in a large number of observed variants or species (e.g., see, Bhattacharya et al. 2007), which may be mistaken for strong evidence of coevolution if the phylogeny is not accounted for. Substitution models provide a probabilistic framework for modeling of both phylogenetic relationships and underlying substitution processes.

In this article, we introduce MESSI (Modeling the Evolution of Secondary Structure Interactions), a probabilistic model that generalizes upon the pioneering Muse (1995) (M95) model of base-pairing evolution. The first way we extend the M95 model is the addition of parameters that allow us to differentiate between rates of evolution affecting the three canonical base-pairs. We used this to compare the role of GU base-pairs in single-stranded RNA viruses with GT base-pairs in single-stranded DNA viruses.

It is well-established that GU pairs can hydrogen bond in RNA to form base-pairs, although they are chemically weaker than GC and AU base-pairings (Rousset et al. 1991). The relative chemical strengths of GC, AU, and GU base-pairs are partially due to the number of hydrogen bonds that form between their constituent bases: three for GC base-pairs, two for AU base-pairs, and two for GU base-pairs. Although GU pairs form the same number of hydrogen bonds as in AU pairs, the geometry of the bases leads to the GU pairing being substantially weaker than the AU pairing (Varani and McClain 2000). Despite the weaker chemical interaction, GU base-pairings are known to be involved in functional RNA structures (Gautheret et al. 1995). Less well understood is the role of GT base-pairings in DNA. There are few reports of GT base-pairings in double-stranded DNA helices (Early et al. 1978; Ho et al. 1985). Although we were unable to directly measure the chemical strength of these base-pairing interactions in the present study, we used MESSI to analyze alignments for evidence of evolutionary forces favoring GT pairs at base-paired positions.

The second way in which we extended the M95 model was to allow substitution rates to vary across sites (Yang 1993, 1994), including allowing the two positions involved in a base-pairing to each to have a potentially different substitution rate. This was done to account for site-specific substitution rates, such as those expected within coding sequences. This is particularly important for virus genomes, where the majority of nucleotides are in protein coding regions, where some of these nucleotides additionally participate in functionally important base-pairing interactions.

The third extension was to permit the strength of coevolution to vary across base-paired sites. This provides a measure of base-pairing coevolution between every pair of sites in alignment, allowing us to test whether a particular pair of sites is coevolving in a manner favoring canonical base-pairing, or whether the two sites are evolving independently of one another. The use of an evolutionary model addresses the problem of founder effects potentially inflating signals of covariation. We used this extension to estimate rates of coevolution at individual base-paired sites within two HIV alignments, allowing us to identify and rank substructures within the larger HIV genomic secondary structure that have potential biological functionality. This is a feature of our model that we expect will assist researchers in focusing their experimental analyses on those portions of large RNA or DNA secondary structures that are most likely to be biologically relevant.

Compared with nonevolutionary methods, the computational cost of applying evolutionary models, such as MESSI, can severely limit their utility. We used graphics processing unit (GPU) parallelism and a Metropolis-within-Gibbs procedure when performing Bayesian inference to reduce these computational bottlenecks. This provided large speed-ups. Furthermore, this allowed us to account for a potentially unknown secondary structure configuration, while simultaneously estimating parameters of interest. This implies that the user need not provide a secondary structure as input. Relying on a potentially incorrect input secondary structure may bias parameter estimates, and may also undermine the conclusions of hypothesis tests based on those estimates. A further benefit of accounting for an unknown secondary structure is that this enables MESSI to output a prediction of the secondary structure and a base-pairing probability matrix.

## Results

### Site Permutation Benchmarks

To assess the degree to which secondary structure dependencies present in real data sets influence model fit, ML inference was performed on real and permuted data sets, and their structure-integrated likelihoods and structure information entropies were compared (see “Site Permutations” in Materials and Methods). The structure-integrated likelihoods for the permuted data sets were expected to be lower than those of the real data sets. Note that comparing these likelihoods is valid given they are in effect marginal likelihoods. Conversely, the structure information entropies were expected to be higher for the permuted data sets than for the real data sets. Unlike the real data sets, the patterns of coevolution in the permuted data sets were not expected to coincide with stable secondary structure configurations, thereby spreading the probability mass over a larger number of secondary structure configurations.

The maximum likelihood estimates of the structure-integrated likelihoods were indeed lower for the permuted data sets in every instance (supplementary table S2, Supplementary Material online). This partially validates our model and is consistent with the presence of real secondary structure dependencies in the original data sets. As expected, the structure information entropies were higher for the permuted data sets, with the exception of RF00003, which had marginally lower structure information entropies for both of the permuted data sets. This result is surprising as RF00003 corresponds to the U1 spliceosomal RNA, a component of a spliceosome (Burge et al. 2013) with a thermodynamically stable structure. Since MESSI uses evolutionary and not thermodynamic information to infer secondary structure, one explanation may be that the patterns of nucleotides within the RF00003 data set are only weakly informative of the underlying secondary structure.

### Benchmarks of RNA Structure Prediction

Although our model was not designed to predict RNA secondary structure, the expected base-pairing and unpairing probabilities can be calculated (see supplementary section 1.5, Supplementary Material online) and a Maximum Expected Accuracy consensus secondary structure determined (see supplementary section 1.6, Supplementary Material online). Our method was compared with two comparative methods of RNA secondary structure prediction: RNAalifold (Bernhart et al. 2006) and PPfold (Sükösd et al. 2012). The three methods were benchmarked on 99 alignments each having a corresponding experimentally determined canonical RNA secondary structure from the RFAM database (Burge et al. 2013). Five different measures were used to compute predictive accuracy (see supplementary section 1.8, Supplementary Material online, for definitions of these measures).

**Fig. 1.**
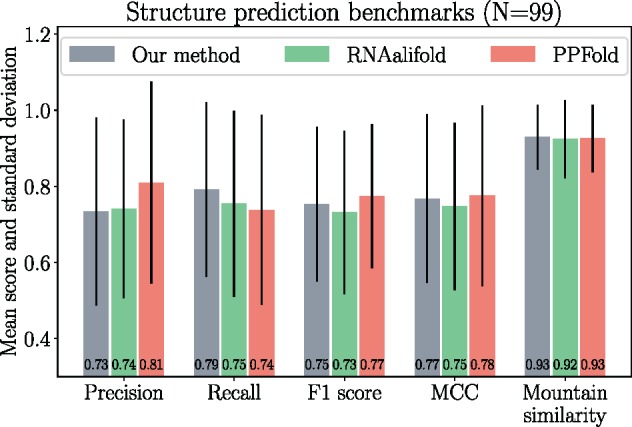
Summary of secondary structure prediction benchmarks. Structure predictions were performed on 99 RFAM data sets using three different comparative structure prediction methods (MESSI, RNAalifold, and PPFold).

MESSI has lower precision but higher recall than the other two methods, implying that it predicts more base-pairs (higher recall), but with a higher number of false-positives (lower precision; fig. 1). For the F1-score and MCC measures, both of which combine precision and recall, MESSI performs slightly better than RNAalifold, and slightly worse than PPfold. MESSI performs marginally better with respect to the mountain similarity measure—a measure that takes into account the overall “shape” of the secondary structures being compared, rather than the exact matching of base-pairs.

Overall, our method performs on a par with two well-established methods of comparative RNA structure prediction. This was surprising given that the model was not developed for the purpose of secondary structure prediction. Maximum likelihood inference was used to estimate the model parameters. Where the coevolution parameters ($\lambda_{\text{GC}},\;\lambda_{\text{AU}}$, and $\lambda_{\text{GU}}$) were free to vary with the only restriction being: $\lambda_{\text{GC}}\geq 1,\;\lambda_{\text{AU}}\geq 1$, and $\lambda_{\text{GU}}\geq 1$. Although not tested here, it might be possible to improve the predictive accuracy of MESSI’s structure predictions by performing Bayesian or MAP inference of the parameters using a set of priors whose hyperparameters are determined from a training data set of alignments and corresponding structures.

### CPU and GPU Timing Benchmarks

The two computational bottlenecks in performing both maximum likelihood and Bayesian inference are computing paired site likelihood matrices (computed using an iterative version of Felsenstein’s algorithm) and computing inside probability matrices (using an iterative version of the inside algorithm); both of these steps are required repeatedly. Although optimized CPU implementations written in Julia were created for both of these steps, these were still relatively slow. Therefore, GPU implementations written in CUDA were implemented for both.

**Fig. 2.**
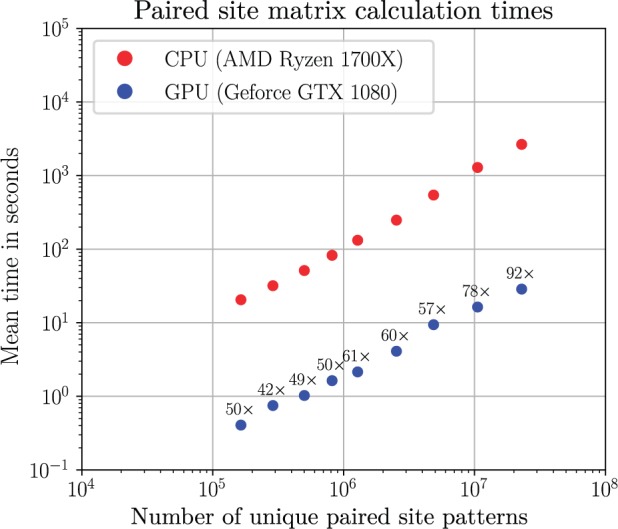
Paired site likelihoods calculation timings in seconds (log_10_ axis) as a function of the number unique paired partial site patterns (log_10_ axis). Numbers above the GPU timings indicate the fold speed-up over the CPU version.

The number of computational steps is expected to grow linearly with the number of unique paired site patterns and hence this was chosen as a predictor of the computational time required (fig. 2). Compared with the single-threaded CPU implementation, we achieve a ∼50× speed-up with the GPU implementation across most data sets.

**Fig. 3.**
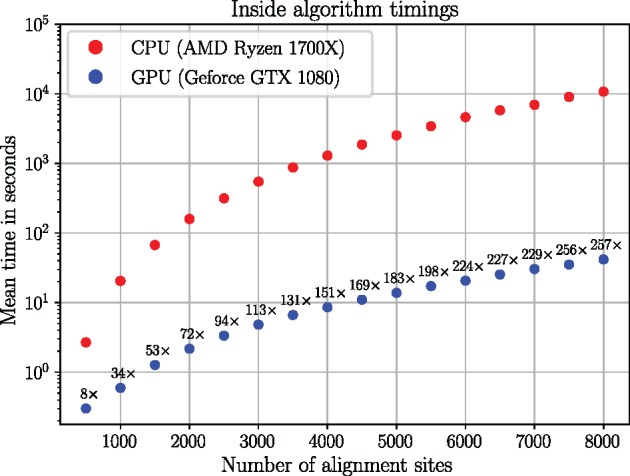
Inside algorithm timings in seconds (log_10_ axis) as a function of the number of alignment sites. Numbers above the GPU timings indicate the fold speed-up over the CPU version.

The number of computational steps for the inside algorithm is expected to grow $O(L^{3})$ where *L* is the number of alignment sites (fig. 3). A 50- to 200-fold speed-up for the paired site likelihood calculations was achieved for moderate data set sizes, with the fold speed-ups for larger data sets being bigger, due to larger data sets better saturating the GPU.

The speed-ups seen here are significant, enabling us to analyze data sets which would typically be considered intractable. Note that CPU and GPU implementations were also developed for the outside algorithm with similar speed-ups obtained (supplementary fig. S4 in the Appendix, Supplementary Material online).

### The Role of GU and GT Base-Pairs in Single-Stranded RNA and DNA

For all five noncoding RNA data sets (RF00001, RF00003, RF00010, RF00379, and RF01846), likelihood ratio tests (LRTs) rejected the GU neutral model in favor of the unconstrained model (*P *<* *0.0005. See table 1). This was true for both the potentially recombinant and the recombination-free data sets. This is evidence that many GU pairs are under selective maintenance in the five noncoding RNA data sets tested.

**Table 1.** Tests of the GU/GT Neutral Hypothesis across 15 Data Sets: Five Noncoding RNA Alignments from the RFAM Database (denoted by the prefix “RF”), Five ssRNA Virus Alignments (foot-and-mouth disease, human poliovirus 1, tobamovirus, rhinovirus A, and hepatitis A virus), and Five ssDNA Virus Alignments (maise streak virus, tomato yellow leaf curl virus, beet curly top virus, and wheat dwarf virus).

Data Set	Type	Number of Sites	Potentially Recombinant			Recombinant Regions Separated			
			LRT (M1-M0)		${\hat{\lambda }}_{\text{GU}}$	LRT (M1-M0)		Bootstrap	${\hat{\lambda }}_{\text{GU}}$
			ΔLL	*P-*Value	(${\hat{\lambda }}_{\text{GT}}$)	ΔLL	*P-*Value	*P-*Value	(${\hat{\lambda }}_{\text{GT}}$)
RF00001	ncRNA	230	226.80	***	2.15	211.41	***	* (P<0.05)	2.17
RF00003	ncRNA	203	46.57	***	2.57	43.51	***	* (P<0.05)	2.56
RF00010	ncRNA	996	2,964.36	***	2.35	797.29	***	* (P<0.05)	2.32
RF00379	ncRNA	335	38.78	***	1.97	35.31	***	* (P<0.05)	1.96
RF01846	ncRNA	624	101.72	***	2.18	71.74	***	* (P<0.05)	2.10
FMDV	ssRNA	8,349	336.64	***	2.75	211.53	***	n.c.	2.50
Hepatitis A	ssRNA	7,572	2.33	*	1.28	3.27	*	n.c.	1.30
H. poliovirus 1	ssRNA	7,668	132.18	***	3.52	140.32	***	n.c.	3.38
Rhinovirus A	ssRNA	7,308	2,255.91	***	10.63	2,188.94	***	n.c.	8.70
Tobamovirus	ssRNA	6,849	90.81	***	2.17	86.96	***	n.c.	2.23
BCTV	ssDNA	3,215	0.18	n.s.	1.08	0.10	n.s.	n.s. (P≈0.77)	1.06
Bocavirus	ssDNA	5,577	0.00	n.s.	1.00	0.00	n.s.	n.c.	1.00
MSV	ssDNA	2,755	3.77	*	1.35	0.00	n.s.	n.s. (P≈0.92)	1.00
TYLCV	ssDNA	2,925	4.12	**	1.50	0.00	n.s.	n.s. (P≈0.87)	1.00
WDV	ssDNA	2,755	0.04	n.s.	1.04	0.00	n.s.	n.s. (P≈0.87)	1.04

note.—n.s., not significant; n.c., not computable.

* *P* < 0.05;

** *P* < 0.005;

*** *P* < 0.0005.

For four of the five RNA virus data sets tested (Rhinovirus A, Tobamovirus, human poliovirus 1, and foot-and-mouth disease virus, see table 1) LRTs rejected the GU neutral model in favor of the unconstrained model (*P *<* *0.0005 in all four cases). Curiously, the GU neutral model could not be rejected in favor of the unconstrained model for the hepatitis A virus data set (table 1), with the ML estimate for ${\hat{\lambda }}_{\text{GU}}=1$. The pattern of results was the same for both the potentially recombinant and the recombination-free data sets.

Three of the five DNA virus genome data sets tested (Human bocavirus, beet curly top virus, and tomato yellow leaf curl virus in table 1) showed no significant difference between the unconstrained model and a GU (GT) neutral model ($\lambda_{\text{GU}}:=1$). In contrast, the Wheat Dwarf Virus data set rejected the GT neutral model (*P *<* *0.05), and the maise streak virus data set rejected the GT neutral model (*P *<* *0.005). ML estimates for ${\hat{\lambda }}_{\text{GT}}$ were in the range 1.0–1.50 for the five ssDNA virus data sets, which was low compared with those determined for the noncoding RNA and RNA virus data sets. Interestingly, for the recombination-free data sets, the GT neutral model could not be rejected for all five data sets, including all four cases where bootstrap *P*-value calculations were performed. The ML estimates for ${\hat{\lambda }}_{\text{GT}}$ were in the range 1.0–1.06 when accounting for recombination.

The LRT results and the ML estimates for ${\hat{\lambda }}_{\text{GU}}$ (${\hat{\lambda }}_{\text{GT}}$) suggest that GT pairs are under weak selective maintenance in DNA virus genomes, and strong selective maintenance in RNA virus genomes and noncoding RNAs. This may indicate that GT base-pairings in DNA are chemically weaker relative to GU base-pairings in RNA and hence do not stabilize DNA secondary structures to the same extent as GU base-pairings in RNA.

Please note that these analyses were analyzed under a model without variable degrees of coevolution (see “Modeling Variable Degrees of Coevolution”). This was done to ensure stable convergence of the optimization algorithm when performing ML inference, which is important for valid LRT statistics and to reduce the computational burden (which scales with number of coevolution categories, the default being five categories, whereas one category was used in this case).

### Relative Coevolution Rates

The relative selective strengths of the coevolution rates associated with GC, AU, and GU pairs were compared across both DNA and RNA virus genomes. The original M95 model assumed that $\lambda_{\text{GC}}:=\lambda_{\text{AU}}$ and $\lambda_{\text{GC}}:=1$. Experimental evidence confirms theoretical predictions of physical chemistry that GC base-pairings are stabilized more by hydrogen bonds than AU base-pairings in RNA (Mathews et al. 1999), with both being substantially stronger than GU base-pairings.

To assess whether $\lambda_{\text{GC}}:=\lambda_{\text{AU}}$ is a reasonable assumption, we performed LRTs comparing the unconstrained model to a $\lambda_{\text{GC}}:=\lambda_{\text{AU}}$ constrained model. For 14 of the 15 data sets, LRTs rejected the GC–AU constrained model in favor of the unconstrained model (results not shown). The only exception was the human poliovirus 1 data set, where the GC–AU constrained model could not be rejected.

This was explored further by comparing the inferred relative magnitudes of the rates associated with GC, AU (AT), and GU (GT) dinucleotides. If the fitness value of a RNA secondary structure element is positively correlated with its chemical stability, it is expected that the relative chemical stabilities associated with the three canonical base-pairs would be reflected in the relative magnitudes of the coevolution rates inferred by MESSI.

**Fig. 4.**
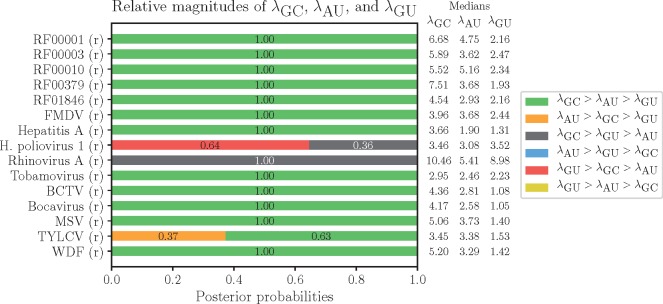
Estimated posterior probabilities for all six orderings of the three base coevolution rates across 15 data sets.

We applied MESSI’s Bayesian posterior inference mode to 15 data sets, five from each of three data set types. Posterior probabilities associated with all six possible orderings of the three base coevolution rates were estimated for each data set (fig. 4). Given the relative chemical base-pairing stabilities, the dominant ordering for the base coevolution rates was expected to be $\lambda_{\text{GC}}>\lambda_{\text{AU}}>\lambda_{\text{GU}}$. For all five ncRNA data sets, all five ssDNA virus data sets, and two of the five ssRNA virus data sets the posterior probability associated with the $\lambda_{\text{GC}}>\lambda_{\text{AU}}>\lambda_{\text{GU}}$ ordering was indeed 1.0.

Interestingly, an unexpected ordering, $\lambda_{\text{GC}}>\lambda_{\text{GU}}>\lambda_{\text{AU}}$, emerged for two of the ssRNA with a posterior probability of 1.0 for the Rhinovirus A data set and posterior probability of 0.36 the Human poliovirus 1 data set. An alternative ordering for the Human poliovirus 1 data set was $\lambda_{\text{GU}}>\lambda_{\text{GC}}>\lambda_{\text{AU}}$ with a posterior probability of 0.64. The ssDNA virus TYLCV data set also displayed the ordering $\lambda_{\text{AU}}>\lambda_{\text{GC}}>\lambda_{\text{GU}}$ with a posterior probability of 0.37. Possible explanations for this result include: 1) for many data sets it is not valid to assume a canonical secondary structure that is conserved across the entire phylogeny (Rivas et al. 2017). Additionally, the secondary structure may be in the form of genome-scale ordered RNA structure (Simmonds et al. 2004) which is not expected to be conserved. Two or more parts of the phylogeny may have different mutually exclusive secondary structures, giving rise to misleading patterns of pair evolution, and 2) data sets with coding regions have additional constraints on synonymous and nonsynonymous substitutions, and these protein coding constraints might mislead MESSI.

### Degrees of Coevolution Are Correlated with Experimental SHAPE-MaP Quantities

A notable example of a large RNA structure that has been partially experimentally determined is that of the HIV-1M subtype B NL4-3 isolate (Watts et al. 2009; Siegfried et al. 2014). Rather than relying solely on computational techniques for the determination of RNA secondary structure of the 9,173 nucleotide NL4-3 genome, the hybrid experimental-computational SHAPE-MaP (Selective 2′-hydroxyl acylation analyzed by primer extension and mutational profiling; Siegfried et al. 2014) approach was used to model the structure. The SHAPE-MaP approach preferentially mutates unpaired nucleotides, allowing the mutated nucleotides to be identified using DNA sequencing following reverse transcription. The SHAPE-MaP reactivity information is then used to constrain a thermodynamic RNA folding algorithm, enabling the construction of a secondary structure model which is reflective of the experimental data.

We compared three nonevolutionary computational measures of covariation (A. Mutual information, B. RNAalifold mutual information, and C. Mutual information with stacking; Lindgreen et al. 2006) and two evolutionary measures of coevolution inferred by MESSI (D. Posterior probability η≠1, and E. Posterior mean *η*) with experimental SHAPE-MaP reactivities and SHAPE-MaP pairing probabilities at base-paired sites corresponding to three different data sets: an HIV 1 b data set, an HIV group 1 M data set, and a Simian Immunodeficiency Virus (SIV) data set. When analyzing the HIV data sets the SHAPE-MaP reactivities, SHAPE-MaP pairing probabilities and base-pairings were derived from a SHAPE-MaP analysis of the HIV NL4-3 sequence (Watts et al. 2009). When analyzing the SIV data set a SHAPE-MaP analysis of the SIVmac239 sequence (Pollom et al. 2013) was used. Given that high SHAPE-MaP reactivities indicate unpairing, we expected that degrees of coevolution (or covariation) would be negatively correlated with SHAPE-MaP reactivities. Conversely, given that some paired nucleotides are expected to be selectively maintained due to structure-related functional importance, we expected a positive correlation between degrees of coevolution (or covariation) and SHAPE-MaP pairing probabilities.

For all three data sets, the two measures of coevolution (D and E) were significantly correlated with both the SHAPE-MaP reactivities and SHAPE-MaP pairing probabilities using Spearman’s rank correlation test. The correlations were in the expected direction (negatively correlated for SHAPE-MaP reactivities and positively correlated for SHAPE-MaP pairing probabilities; table 2). For all three data sets, the correlation coefficients were significantly stronger in the expected direction for the two coevolution measures (D and E) than the three covariation measures (A, B, and C; see the 95% confidences intervals for Spearman’s rho). We note that although many of the correlations were statistically significant, the magnitudes of the correlations were weak.

**Table 2.** Spearman’s Correlations (*ρ*) and 95% Confidence Intervals (*ρ* 95% CI) between Five Different Measures of Covariation/Coevolution and Base-Pair Averaged SHAPE-MaP Reactivities and the Same Five Measures and Base-Pair Averaged SHAPE-MaP Pairing Probabilities.

Data Set	Measure	SHAPE-MaP Reactivities	ρ 95% CI	*P-*Value	SHAPE-MaP Pairing Probabilities	ρ 95% CI	*P-*value
		ρ			ρ		
	A. Mutual information (MI)	−0.01	[−0.051, 0.035]	n.s.	0.11	[0.069, 0.154]	***
	B. RNAAlifold MI	0.01	[−0.033, 0.053]	n.s.	0.01	[−0.030, 0.056]	n.s.
HIV-1	C. MI with stacking	−0.02	[−0.060, 0.026]	n.s.	0.10	[0.059, 0.144]	***
Subtype B	D. p(η≠0)	−0.16	[−0.202, −0.118]	***	0.19	[0.147, 0.230]	***
	E. Posterior mean *η*	−0.14	[−0.180, −0.095]	***	0.20	[0.162, 0.244]	***
	A. Mutual information (MI)	0.03	[−0.016, 0.070]	n.s.	0.09	[0.047, 0.132]	***
	B. RNAAlifold MI	0.03	[−0.013, 0.073]	n.s.	0.08	[0.033, 0.119]	***
HIV-1	C. MI with stacking	0.00	[−0.043, 0.043]	n.s.	0.12	[0.081, 0.166]	***
Group 1M	D. p(η≠0)	−0.18	[−0.225, −0.142]	***	0.27	[0.227, 0.307]	***
	E. Posterior mean *η*	−0.16	[−0.197, −0.113]	***	0.29	[0.251, 0.330]	***
	A. Mutual information (MI)	0.09	[0.053, 0.132]	***	−0.04	[−0.084, −0.005]	*
	B. RNAAlifold MI	0.12	[0.086, 0.164]	***	−0.07	[−0.114, −0.035]	***
SIVmac239	C. MI with stacking	0.10	[0.057, 0.135]	***	−0.01	[−0.046, 0.033]	n.s.
	D. p(η≠0)	−0.12	[−0.160, −0.082]	***	0.19	[0.153, 0.229]	***
	E. Posterior mean *η*	−0.10	[−0.137, −0.058]	***	0.20	[0.164, 0.240]	***

Note.—Underlined values indicate correlations that are statistically significant and in the expected direction. n.s., not significant.

* *P* < 0.05;

** *P* < 0.005;

*** *P* < 0.0005

Curiously, for the SIV data set, SHAPE-MaP reactivities were significantly positively correlated with the three measures of covariation (A, B, and C) rather than negatively correlated as expected. There is broad evidence to suggest that base-paired sites in a functionally important RNA structure tend to be more conserved (less variable) due to being under selective constraint (Tuplin et al. 2004; Muhire et al. 2014) and that double-stranded RNA (i.e., base-paired positions) is less susceptible to mutational processes (Lindahl and Nyberg 1974). Conversely, unpaired sites are expected to undergo relatively higher rates of mutation. These higher rates of mutation may cause the three nonevolutionary measures of covariation to be erroneously inflated, given that they do not fully account for site-to-site rate variation (see supplementary section 1.2, Supplementary Material online) unlike the coevolution measures inferred under our model, which do. It should also be noted that the SIV data set is highly diverse compared with the two HIV data sets. Given these factors, it is anticipated that weakly base-paired sites will have inflated degrees of covariation using measures A, B, and C, which may explain the unexpected positive correlation.

Overall, these results provide some reassurance that our method is performing as expected and that the evolutionary measures of coevolution are more reliable than the three measures of covariation that do not take into account evolutionary dependencies among the sequences being analyzed. The detected degrees of coevolution suggest that a large proportion of the predicted base-pairings in the SHAPE-MaP structures have been selectively maintained since the common ancestors of the sequences being analyzed in each of the three data sets.

### Ranking and Visualization of Substructures

Rather than considering the entire secondary structure of a large sequence, it is often useful to consider individual substructures. There are two primary reasons for considering substructures: 1) smaller regions are more easily conceptualized, and 2) if functional components of a secondary structure are present, they tend to correspond to small regions (20–350 nucleotides long) of that secondary structure.

MESSI automatically ranks substructures by degrees of coevolution between their constituent nucleotides (see supplementary methods section 1.9, Supplementary Material online). We produced two rankings based on an HIV-1 subtype B alignment. The first ranking treated the HIV-1 NL4-3 SHAPE-MaP secondary structure as the canonical structure when inferring coevolution and identifying substructures (denoted the SHAPE structure ranking; table 3 and supplementary table S5, Supplementary Material online). The second ranking used a consensus structure estimated by MESSI based on base-pairing probabilities (denoted the consensus structure ranking; table 4 and supplementary table S6, Supplementary Material online).

**Table 3.** SHAPE Structure Ranking.

Rank	Alignment Position	NL4-3 Position	Length	Name	Median Degree of Coevolution	*z-*Score
1	8233–8582	7249–7595	350	Rev response element (RRE)	5.38	5.02
2	2608–2943	1991–2326	336	Longest continuous helix	5.17	2.92
3	10155–10383	8982–9170	229	3′-Untranslated region (3′-UTR)	5.27	2.69
4	588–838	105–344	251	5′-Untranslated region (5′-UTR)	5.65	2.61
5	9570–9584	8440–8454	15		5.91	2.29
6	860–979	366–485	120	5′-Untranslated region (5′-UTR)	5.54	2.28
7	1710–1845	1177–1312	136		5.17	2.28
8	2115–2301	1561–1711	187	Gag-pol frameshift	5.31	2.21
9	1479–1490	946–957	12		5.85	2.04
10	3886–3907	3269–3290	22		5.80	2.01

note.—The top 10 of 86 nonoverlapping HIV NL4-3 substructures ranked from highest to lowest *z*-score based on the estimated degrees of coevolution within an alignment of HIV-1 subtype B sequences. Where the HIV NL4-3 SHAPE-MaP secondary structure was used as the canonical structure.

**Table 4.** Consensus Structure Ranking.

Rank	Alignment Position	NL4-3 Position	Length	Name	Median Degree of Coevolution	*z*-Score
1	8240–8577	7256–7590	338	Rev response element (RRE)	5.64	6.53
2	2202–2229	1645–1672	28	Gag-pol frameshift	8.17	4.56
3	1710–1845	1177–1312	136		6.44	4.50
4	4751–4833	4134–4216	83		6.47	3.97
5	4505–4709	3888–4092	205		5.22	3.21
6	591–939	108–445	349	5′-Untranslated region (5′-UTR)	5.38	3.16
7	133–151	NA	19		6.85	2.94
8	2564–2890	1947–2273	327	Longest continuous helix	4.44	2.62
9	9782–9800	8645–8663	19		6.92	2.55
10	3612–3623	2995–3006	12		6.74	2.50

note.—The top 10 of 118 nonoverlapping HIV consensus substructures ranked from highest to lowest *z*-score based on their degrees of coevolution within an alignment of HIV-1 subtype B sequences. Where the canonical structure was treated as unknown and a consensus structure predicted by MESSI.

**Fig. 5.**
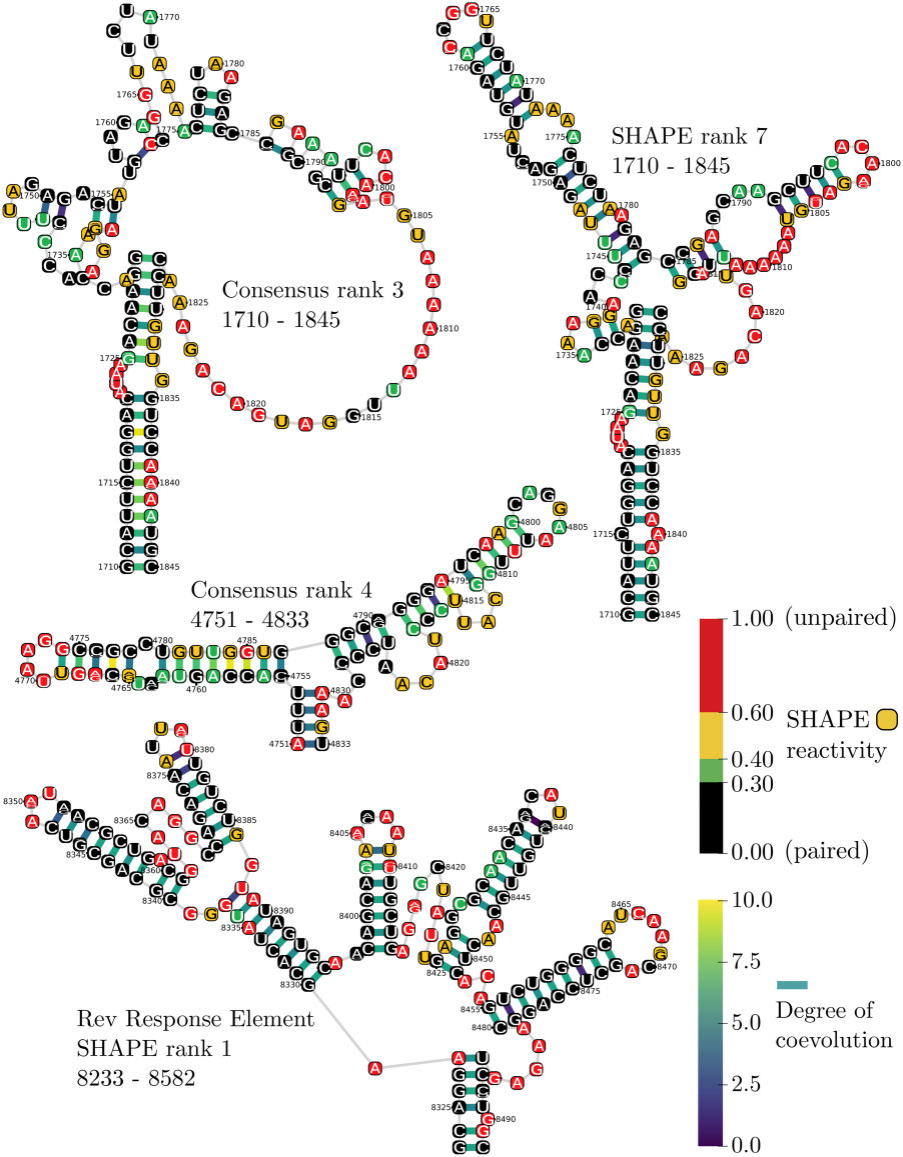
Visualization of several top ranking substructures in the SHAPE-MaP structure and consensus structure rankings. NL4-3 SHAPE-MaP experimental reactivities are mapped and visually overlaid using the same color scheme as in Watts et al. (2009). Depicted within each nucleotide is a sequence logo summarizing the nucleotide composition at the corresponding alignment position. Mean degrees of coevolution inferred using MESSI are depicted for each base-pair using colored links (blue–green–yellow gradient).

The highest ranked substructure in both the SHAPE and consensus rankings was the RRE (SHAPE RRE visualized in fig. 5). The RRE occurs in the genomes of all known HIV groups and plays a crucial role in the regulation of HIV virion expression (Heaphy et al. 1990; Daugherty et al. 2010).

The longest continuous helix identified in both the SHAPE-MaP and MESSI structures was ranked 2nd in the SHAPE ranking and 8th in the consensus ranking, respectively. The SHAPE-MaP analysis revealed that this helix is highly stable, although its function is unknown. The significant degrees of coevolution detected at base-paired sites within this substructure and the fact that MESSI detects it as conserved across all HIV-1 subtype sequences provides further evidence of its likely functional importance.

Portions of the 3′- and 5′-untranslated regions (UTRs) were ranked 3rd and 4th in the SHAPE ranking, respectively. This was not surprising given that these are both noncoding regions. The 5′-UTR is involved in regulation of translation (Damgaard et al. 2004), whereas the 3′-UTR is believed to be involved in regulation of transcription (Watts et al. 2009). A 5′-UTR substructure at a similar position is ranked 6th in the consensus ranking, whereas a 3′-UTR substructure at a similar position was not detected in the consensus structure. This may be explained by the large number of UTR missing sequences and high degrees of alignment uncertainty in the HIV-1 subtype B alignment in the UTR regions; factors which would both reduce support for the predicted base-pairings in the consensus structure.

An uncharacterized substructure (alignment position: 1710–1845) ranked 7th in the SHAPE structure ranking and 3rd in the consensus structure ranking (fig. 5). This substructure warrants further study, given the supporting evidence from experimental SHAPE-MaP reactivities, MESSI’s coevolution estimates, and MESSI’s evidence of conservation across HIV-1 subtype B sequences. Despite MESSI predicting the same helix as SHAPE-MaP at the base of this substructure, the remainder of the substructure is different in the SHAPE-MaP model. It is likely that the SHAPE-MaP model of this substructure is more accurate in this instance.

Interestingly, an additional uncharacterized substructure (alignment position: 4751–4833) ranked 4th in consensus ranking, but was not present in the HIV-1 NL4-3 SHAPE structure and hence was not present in the SHAPE structure ranking (fig. 5). Overlaid SHAPE-MaP reactivities from the HIV N4L-3 SHAPE model provide some support for MESSI’s prediction; particularly at unpaired positions which are supported by high SHAPE-MaP reactivities (indicating single-strandedness). It is possible that either MESSI’s or SHAPE-MaP’s prediction is wrong, or that the particular conformation predicted by MESSI is conserved among a subset of HIV-1 subtype B sequences that excludes NL4-3. It is also possible that this substructure exists in alternative conformations depending on in vivo or in vitro conditions.

Finally, the gag-pol frameshift-associated substructure was ranked 8th in the SHAPE ranking and 2nd in the consensus rankings. This substructure regulates the ratio of HIV gag/gag-pol that is expressed. Ribosomal synthesis of the gag-pol polyprotein requires a −1 ribosomal frameshift, without which translation ends in synthesis of the gag protein alone.

Overall, there is an excess of top-ranking substructures that have been identified previously in the literature as having structure-related importance. This is particularly evident in the SHAPE-MaP structure ranking. The use of the experimentally determined SHAPE-MaP structure as the canonical structure strongly informs the SHAPE structure ranking, but has the disadvantage that it is based only on the HIV NL4-3 sequence rather than being representative of base-pairings conserved across all sequences within the HIV-1 subtype B alignment. By contrast, the consensus ranking canonical structure is predicted by MESSI and is based solely on evolutionary information, rather than experimental or thermodynamic information. In the future, we hope to extend MESSI by adding both experimental constraints from experiments such as SHAPE-MaP and thermodynamic constraints from folding software such as Vienna RNAfold. We expected this to improve estimates of coevolution and the overall ranking provided by MESSI.

## Concluding Remarks

MESSI was developed for modeling substitutions that are consistent with the maintenance of canonical base-pairing at paired sites within alignments of DNA and RNA sequences. To achieve this, we extended an existing model, M95 (Muse 1995), in four major ways: 1) differentiating between the three canonical base-pairs (GC, AU, and GU), 2) allowing substitution rates to vary across sites, 3) permitting the strength of coevolution to vary across base-paired sites, to measure the strength of selection operating on particular base-pairs, and 4) accounting for a potentially unknown secondary structure.

Among these extensions, extending the model to permit an unknown secondary structure posed the greatest computational challenges. The first challenge was the need to compute likelihoods using Felsenstein’s peeling algorithm for all $\left(\begin{array}{c} L\\ 2 \end{array}\right)$ paired sites. Fortunately, a large number of redundant calculations could be avoided due to a large proportion of paired sites sharing the same partial site patterns (Pond and Muse 2004), resulting in at least a 5× speed-up. Additionally, a further 50× speed-up was achieved using a GPU implementation of Felsenstein’s peeling algorithm. The second challenge was the need to marginalize an unknown secondary structure using the inside algorithm. Computational speed-ups of 50×–200× were achieved using a GPU implementation of the inside algorithm. For Bayesian inference, a Metropolis-within-Gibbs procedure was implemented to further avoid calculating the paired matrix likelihoods and inside probabilities at every iteration.

ML and Bayesian inference were used for different analyses. ML inference allowed us to perform likelihood ratio tests of various hypotheses, for which Bayesian model comparison was computationally intractable. Bayesian inference was used to obtain posterior distributions over various parameters, including the rates of coevolution associated with the three canonical base-pairs, and posterior probabilities and degrees of coevolution at base-pair sites.

To perform an initial validation of our model, site permutations of nucleotide alignments were performed to disrupt the secondary structure dependencies expected in real data sets. Consistent with the model behaving desirably, the structure-integrated maximum likelihood values were lower, and the structure information entropy values higher for the permuted data sets overall.

The ability to marginalize an unknown secondary structure shared among an alignment of sequences, implies that MESSI is also capable of secondary structure prediction. Although MESSI was not designed with structure prediction in mind, we found that it performed similarly to two popular comparative secondary structure prediction methods: RNAalifold (Hofacker 2009) and PPfold (Sükösd et al. 2012). This result further validates our approach.

We found strong evidence that GU pairs are selectively favored at base-paired sites in five noncoding RNA data sets and four of five RNA virus genome data sets. Strong evidence for selection of GT pairs at base-paired sites was found for only one out of five of the DNA virus data sets tested. Whereas no evidence was found when taking into account recombination. The notion that GU pairs play a role in stabilizing RNA secondary structures is consistent with numerous phylogenetic, and experimental analyses of RNA molecules (Woese et al. 1980; Eddy and Durbin 1994; Deigan et al. 2009). The role of GT base-pairings in stabilizing DNA genomic secondary structures remains unclear.

We applied our model to the HIV-1 NL4-3 secondary structure and two corresponding alignment data sets containing large numbers of HIV-1 sequences, and an SIVmac239 secondary structure and a corresponding alignment of SIV sequences. We found that correlations between the SHAPE-MaP-determined quantities and degrees of coevolution as detected using MESSI were stronger than correlations between the same quantities and three nonevolutionary measures of covariation.

Interactive visualizations of the HIV-1 NL4-3 SHAPE-MaP and consensus secondary structures with the inferred degrees of coevolution overlaid were automatically generated by MESSI. Two rankings of substructures based on inferred degrees of coevolution within an alignment of HIV-1 subtype B sequences demonstrated an excess of high-ranking substructures that have been commonly cited in the literature as having structure-related importance. This ranking procedure is expected to aid researchers in characterizing the secondary structures of less well-studied viruses.

A feature that was not fully accounted for in our model and that is especially important for viral genomes, such as HIV, is that their genomes simultaneously encode for proteins. This implies a dual evolutionary constraint, whereby selection may be acting on the amino acid sequence, while simultaneously acting to maintain base-pairing interactions in biologically functional RNA secondary structures. In the future, we would like to consider a model that explicitly accounts for both protein-coding and RNA base-pairing constraints.

A second limitation of our model is the assumption of a canonical RNA secondary structure shared across the entire evolutionary history of the sequences being analyzed. This is considered a reasonable approximation for low- and moderately diverged alignments, where many of the sequences are expected share a high proportion of the same base-pairs. Notwithstanding, it is also likely that different parts of the tree relating the sequences will have at least some parts of those sequence adopting alternative secondary structure conformations. These regions are interesting from a functional perspective. The ability to identify these alternative evolutionary conformations and the mutations responsible for them may lead to significant insights into viral adaptations, such as structural changes following zoonotic transmission of viruses from nonhuman hosts to humans or the development of drug resistance.

## Materials and Methods

### The Muse 1995 Model

Muse (1995) developed a paired site model, henceforth referred to as the M95 model. M95 accounts for RNA base-pairing constraints by modeling pairs of nucleotide positions using a 16×16 matrix. The model generalizes upon standard 4 × 4 nucleotide substitution models, such as the GTR model, by introducing a coevolution parameter, *λ*, that is intended to capture substitutions at paired positions that are consistent with the maintenance of canonical RNA base-pairing. We define the set of canonical base-pairs as follows:

(1) C={GC, CG, AU, UA, GU, UG}

Equation (2) presents a version of the original M95 paired model based on a GTR model *Q* and a set of canonical base-pairs C:

(2) $$M_{\mathrm{ab}}=\{\begin{array}{cc} q_{\mathrm{ab}}\lambda & \text{pairing}\;\text{gained}\;\\ & a\notin C\;\text{and}\;b\in C,e.g.a=\text{AC}\to b=\text{AU}\\ q_{\mathrm{ab}} & \text{pairing}\;\text{unchanged}\;\\ & a,b\notin C\;\text{or}\;a,b\in C,e.g.a=\text{AU}\to b=\text{GU}\\ q_{\mathrm{ab}}/\lambda & \text{pairing}\;\text{lost}\;\\ & a\in C\;\text{and}\;b\notin C,e.g.a=\text{AU}\to b=\text{AC}\\ 0 & 2\;\text{differences}\\ & e.g.a=\text{AU}\to b=\text{GC} \end{array},$$

where *a* and *b* are nucleotide pairs. Note that when *a* and *b* differ at both positions they are assigned a rate of zero. They are only given a positive rate when differing at a single position. Note that *a* and *b* in *q_ab_* refers to the entry of the GTR matrix *Q* corresponding to the differing nucleotide position within the nucleotide pair, and *λ* is a parameter capturing the degree of RNA coevolution; that is, the degree to which canonical RNA base-pairing is evolutionary maintained (λ>1) or disrupted (λ<1). *λ *= 1 represents the *neutral* case, in which each of the two nucleotide positions in a pair are treated as evolving independently under the GTR model specified by *Q*.

Furthermore, let $\pi^{\text{dinuc}}$ denote a length 16 vector of paired frequencies. $\pi^{\text{dinuc}}$ is the concatenation of two mutually exclusive sets: $\pi^{\text{dinuc}}=\pi^{\text{unpaired}⌢}\pi^{\text{paired}},\;\pi^{\text{unpaired}}$ represents the cases where the target pair *c_ij_* is not in the set of canonical base-pairs ($c_{\mathrm{ij}}\notin C$), and $\pi^{\text{paired}}$ represents the cases where the target pair *c_ij_* is in the set of canonical base-pairs ($c_{\mathrm{ij}}\in C$), respectively:

$$\pi_{c_{\mathrm{ij}}}^{\text{unpaired}}=\kappa_{1}^{-1}\pi_{i}\pi_{j}\;\text{and}\;\pi_{c_{\mathrm{ij}}}^{\text{paired}}=\kappa_{1}^{-1}\pi_{i}\pi_{j}\lambda^{2},$$

Note that *i* and *j* correspond to the first and second positions of the target pair, respectively. Where π_*i*_ is the equilibrium frequency under the GTR model, Q, of the nucleotide in the first position of the target pair *c_ij_*, and similarly π_*j*_ is the equilibrium frequency of the nucleotide in the second position. $\kappa_{1}=1+2(\pi_{G}\pi_{C}+\pi_{A}\pi_{U}+\pi_{G}\pi_{U})(\lambda^{2}-1)$ is a normalizing constant that ensures the entries of π^*d*^ sum to 1.

Note that within the set of canonical base-pairs, C (defined in eq. 1), there are three pairs of symmetrical base-pairs: {GC, CG}, {AU, UA}, and {GU, UG}. It is assumed that each base-pair within a symmetrical pair has the same fitness. This is a reasonable assumption as it treats the evolution of nucleotides toward the 5′-end of the sequence the same as nucleotides toward the 3′-end. From this point forward, we assume this symmetry and refer to the three pairs of symmetrical base-pairs as the *three canonical base-pairs*.

In the formulation of the original M95 model in equation (2), all three canonical base-pairs in the set C are treated as having equal fitness. However, there is good evidence that GU base-pairings in RNA, for example, are deleterious evolutionary intermediates relative to GC and AU (Rousset et al. 1991). In light of this, in the next section, we extend the M95 model such that substitutions affecting the three canonical base-pairs are not constrained to have the same rate of coevolution.

### Differentiating between Types of Base-Pairing Substitutions

We extend the M95 model to differentiate between the three different canonical base-pairs, by introducing potentially distinct coevolution rates ($\lambda_{\text{GC}},\;\lambda_{\text{AU}}$, and $\lambda_{\text{GU}}$) for each of three different base-pairs (GC, AU, and GU, respectively). Using similar notation as in equation (2), the extended rate matrix is given as follows:

(3) $$M_{\mathrm{ab}}=\{\begin{array}{cc} q_{\mathrm{ab}}\lambda_{\text{GC}} & \text{GC}\;\text{pairing}\;\text{gained}\\ & e.g.a=\text{AC}\to \;b=\text{GC}\\ q_{\mathrm{ab}}\lambda_{\text{AU}} & \text{AU}\;\text{pairing}\;\text{gained}\\ & e.g.a=\text{AC}\to \;b=\text{AU}\\ q_{\mathrm{ab}}\lambda_{\text{GU}} & \text{GU}\;\text{pairing}\;\text{gained}\\ & e.g.a=\text{GA}\to \;b=\text{GU}\\ q_{\mathrm{ab}}/\lambda_{\text{GC}} & \text{GC}\;\text{pairing}\;\text{lost}\\ & e.g.a=\text{GC}\to \;b=\text{GA}\\ q_{\mathrm{ab}}/\lambda_{\text{AU}} & \text{AU}\;\text{pairing}\;\text{lost}\\ & e.g.a=\text{AU}\to \;b=\text{AG}\\ q_{\mathrm{ab}}/\lambda_{\text{GU}} & \text{GU}\;\text{pairing}\;\text{lost}\\ & e.g.a=\text{GU}\to \;b=\text{GA}\\ q_{\mathrm{ab}}\lambda_{\text{GU}}/\lambda_{\text{GC}} & \text{GC}\;\text{to}\;\text{GU}\\ q_{\mathrm{ab}}\lambda_{\text{GC}}/\lambda_{\text{GU}} & \text{GU}\;\text{to}\;\text{GC}\\ q_{\mathrm{ab}}\lambda_{\text{GU}}/\lambda_{\text{AU}} & \text{AU}\;\text{to}\;\text{GU}\\ q_{\mathrm{ab}}\lambda_{\text{AU}}/\lambda_{\text{GU}} & \text{GU}\;\text{to}\;\text{AU}\\ q_{\mathrm{ab}} & \text{pairing}\;\text{unchanged}\\ & e.g.a=\text{AC}\to \;b=\text{UC}\\ 0 & 2\;\text{differences}\\ & e.g.a=\text{AU}\to \;b=\text{GC}\; \end{array}$$

and the corresponding paired frequencies are:

(4) $$\begin{array}{c} \pi_{c_{\mathrm{ij}}}^{\text{unpaired}}=\kappa_{2}^{-1}\pi_{i}\pi_{j}\\ \pi_{\text{GC}}=\kappa_{2}^{-1}\pi_{G}\pi_{C}\lambda_{\text{GC}}^{2}\\ \pi_{\text{AU}}=\kappa_{2}^{-1}\pi_{A}\pi_{U}\lambda_{\text{AU}}^{2}\\ \pi_{\text{GU}}=\kappa_{2}^{-1}\pi_{G}\pi_{U}\lambda_{\text{GU}}^{2}, \end{array}$$

where $\kappa_{2}=1+2[\pi_{G}\pi_{C}(\lambda_{\text{GC}}^{2}-1)+\pi_{A}\pi_{U}(\lambda_{\text{AU}}^{2}-1)+\pi_{G}\pi_{U}(\lambda_{\text{GU}}^{2}-1)]$.

### Stationarity and Time-Reversibility

We are able show for the extended model that the paired frequencies, π, given in (4) correspond to the stationary distribution of **M** by verifying that:

(5) πM=0,

and that time-reversibility of **M** holds:

(6) $$\begin{array}{cc} \pi_{a}M_{\mathrm{ab}}=\pi_{b}M_{\mathrm{ba}} & \forall_{\mathrm{ab}} \end{array}$$

where *a* and *b* represent nucleotide pairs. The conditions in (5) and (6) were verified using the symbolic math package, SymPy (Joyner et al. 2012), as implemented in the musesymbolic.py script (see Supplementary Material online).

### Modeling Variable Degrees of Coevolution

In the M95 model (2), the rate of coevolution was assumed to be the same for each base-paired site within a secondary structure S. However, it is expected that the strength of the selective forces maintaining canonical base-pairing will vary among base-paired sites in S. In this section, we extend the M95 model such that the *degree of coevolution*, denoted by $\eta_{q,r}$, is able to vary from base-paired site to base-paired site. $\eta_{q,r}$ is drawn independently for each base-paired site (described in the next section), and acts to scale the three coevolution rates as follows:

(7) $$\begin{array}{c} \lambda_{\text{GC}}^{q,r}=(\lambda_{\text{GC}}-1)\eta_{q,r}+1\\ \lambda_{\text{AU}}^{q,r}=(\lambda_{\text{AU}}-1)\eta_{q,r}+1\\ \lambda_{\text{GU}}^{q,r}=(\lambda_{\text{GU}}-1)\eta_{q,r}+1 \end{array},$$

where $\lambda_{\text{GC}}\geq 1,\;\lambda_{\text{AU}}\geq 1$, and $\lambda_{\text{GU}}\geq 1$ are the base-pairing substitution rates shared across all paired sites. This parametrization was chosen so that $\lambda_{\text{GC}}^{q,r}=\lambda_{\text{AU}}^{q,r}=\lambda_{\text{GU}}^{q,r}=1$ when $\eta_{q,r}=0$, where *q*, *r* refer to a pair of nucleotide positions *q* and *r*.

In addition to allowing the degree of coevolution, *η*, to vary across base-paired sites, we also allow substitution rates to vary from site to site following the gamma distributed sites rate approach of (Yang 1993, 1994). For unpaired sites, sequence evolution is modeled using a standard GTR + Γ model. For base-paired sites, slightly more care needs to be taken (see supplementary section 1.2, Supplementary Material online, for details). We call the version of our generalized M95 model that differentiates between the three canonical base-pairs and takes into account site-to-site rate variation, the “unconstrained M95 model.”

### Testing Neutrality of Coevolution

To test the hypothesis that two nucleotide positions within a particular base-paired site are evolving neutrally, that is, the substitutions at each of the two sites are occurring independently rather than actively favoring the maintenance canonical base-pairing, we assume that the degree of coevolution, $\eta_{q,r}$, at each base-paired site is distributed as follows: $\eta_{q,r}=0$ with probability $w_{\eta }$ (the neutral, independent case), otherwise with probability $1-w_{\eta },\;\eta_{q,r}$ is drawn from a discretized gamma distribution with M categories (the dependent case). Note that $\eta_{q,r}\geq 0$ and therefore the case where substitutions are acting to disrupt canonical RNA base-pairing is not considered, that is, the case where the coevolution parameters are between 0 and 1. For all analyses, a discretization of *M *=* *4 was used, resulting in five rate categories: one neutral category with probability $w_{\eta }$, and four positive categories each with probability $\frac{1-w_{\eta }}{4}$.

### Parameters

Table 5 lists the parameters and their distributions used in the most general version of the implemented model (the unconstrained model). Note that for some analyses we perform Bayesian inference, whereas for others, we perform maximum likelihood (ML) inference. The distributions over the parameters specified here are those used for Bayesian inference, however, we also indicate how the parameters are treated during ML inference. Parameters are either estimated while ignoring the prior distribution, or fully marginalized under the prior distribution. Note that the phylogenetic tree, $\hat{T}$, relating the alignment of sequences, D, for both Bayesian and ML inference is estimated in advance and fixed a priori using FastTree (Price et al. 2010) under a GTR+CAT model.

**Table 5.** Parameters of the Unconstrained Model and Their Distributions.

Parameter and Distribution	Marginalized or Estimated	Description
$w_{\eta }∼\text{Beta}(2,2)$	Estimated	Probability of neutral coevolution.
$X_{q,r}∼\text{Bernoulli}(w_{\eta })$	Marginalized	Indicates neutral coevolution at position *q*, *r* when *X_q_*_,__*r*_ = 1.
$c∼\text{Exponential}(\frac{1}{10})$	Estimated	Shape and rate parameter of prior over coevolution rates $\eta_{q,r}$.
$\eta_{q,r}=0\;\text{if}\;X_{q,r}=1$, otherwise:	Marginalized	The rate of coevolution at each paired position *q*, *r*.
$\eta_{q,r}∼{\text{DiscretisedGamma}}_{M}(c,c)$		
$d∼\text{Exponential}(\frac{1}{10})$	Estimated	Shape and rate parameter of prior over substitutions rates *ρ_q_*.
$\rho_{q}∼{\text{DiscretisedGamma}}_{K}(d,d)$	Marginalized	Substitution rate at each unpaired position *q*.
$\left(\pi_{A},\pi_{C},\pi_{G},\pi_{T})∼\text{Dir}(1,1,1,1\right)$	Estimated	GTR equilibrium frequencies of the four nucleotides.
$q_{\text{AC}}∼\text{Exponential}(\frac{1}{10})$	Estimated	GTR rate matrix entry AC.
$q_{\text{AG}}∼\text{Exponential}(\frac{1}{10})$	Estimated	GTR rate matrix entry AG.
$q_{\text{AT}}∼\text{Exponential}(\frac{1}{10})$	Estimated	GTR rate matrix entry AT.
$q_{\text{CG}}∼\text{Exponential}(\frac{1}{10})$	Estimated	GTR rate matrix entry CG.
$q_{\text{CT}}∼\text{Exponential}(\frac{1}{10})$	Estimated	GTR rate matrix entry CT.
$q_{\text{GT}}∼\text{Exponential}(\frac{1}{10})$	Estimated	GTR rate matrix entry GT.
$\lambda_{\text{GC}}∼\text{Exponential}(\frac{1}{10})+1$	Estimated	GC coevolution rate.
$\lambda_{\text{AT}}∼\text{Exponential}(\frac{1}{10})+1$	Estimated	AT coevolution rate.
$\lambda_{\text{GT}}∼\text{Exponential}(\frac{1}{10})+1$	Estimated	GT coevolution rate.
S∼KH99	Marginalized	The secondary structure is drawn from the KH99 SCFG prior.

### Computer Representations of Secondary Structure

To model nucleic acid secondary structure, a suitable definition of secondary structure is required. We use the definition outlined in Moulton et al. (2000): a secondary structure, S, for a nucleic acid molecule consisting of *N* nucleotides is a simple graph specified by the vertex set [N]:={1,…,N} and an edge set $B_{S}$. Where each vertex in [N] corresponds to a nucleotide and each edge in the edge set $B_{S}$ corresponds to a base-pair. S is such that if $\{i,j\},\{k,l\}\in B_{S}$ with *i *<* j* and *k *<* l* then:

1) *i* = *k* if and only if *j* = *l*, and

2) k≤j implies that *i* < *k* < *l* < *j.*

Vertices that are not contained within the edge set $B_{S}$ are termed *unpaired*. Condition (1) implies that each vertex (nucleotide) belongs to at most one base-pair. Condition (2) prevents *pseudoknotting*, that is, nonnested base-pairs.

Note that pseudoknotting is physically possible in both real RNA and DNA structures, but is excluded in many definitions of secondary structures as efficient algorithms exist for marginalizing or maximizing over secondary structures when assuming (2). Our method permits a canonical secondary structure with pseudoknots to be specified a priori, however, if the user instead treats the structure as unknown, MESSI will strictly marginalize over nonpseudoknotted structures only.

**Fig. 6.**
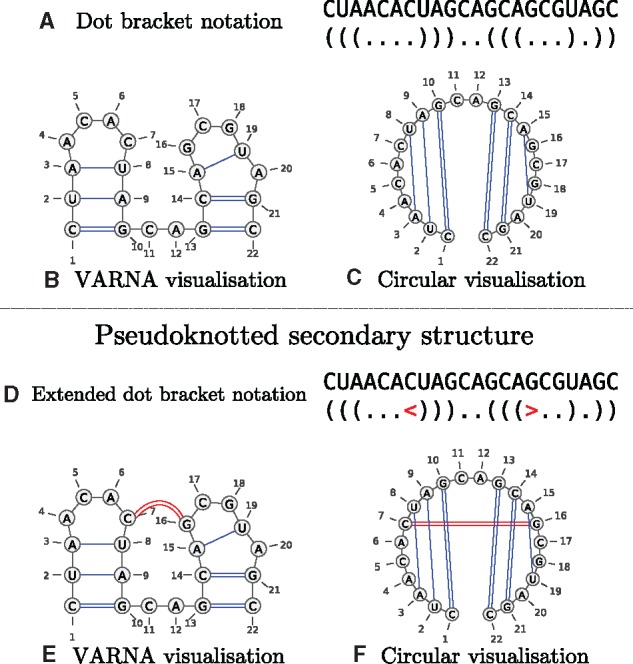
Examples of secondary structure representations. Above (*A*) is a dot bracket representation of a secondary structure, and the corresponding VARNA and circular visualizations (*B* and *C*, respectively) produced by VARNA Darty et al. (2009). Below (*D*) is an extended dot bracket notation format with an additional bracket type, <>, that allows a pseudoknotted structure to be represented unambiguously. (*E*) and (*F*) are the corresponding VARNA visualizations for (*D*). Note how the overlapping bonds in the circular visualization (*F*) demonstrate that the secondary structure is pseudoknotted.

Figure 6 gives a computational format for representing secondary structures. The dot-bracket format (fig. 6*A*) is a natural and compact way of representing nonpseudoknotted secondary structures. Matching brackets represent base-paired nucleotide positions and dots represent unpaired (singled-stranded) nucleotide positions. To represent pseudoknotted structures (structures that violate condition [2]), additional bracket types are required (fig. 6*D*).

### Likelihood

Conditioned on a secondary structure, S, unpaired nucleotide positions within S, denoted by $\overset{\cdot }{q}$, and base-paired nucleotide positions within S, denoted by $\hat{q,r}$, are assumed to be independent. The likelihood of an alignment, D, is given by a simple product of unpaired and paired site likelihoods:

(8) $$p(D|S,\hat{T},\theta )=\overset{\text{unpaired}}{\overset{︷}{\prod_{\overset{\cdot }{q}\in S}p(D_{q}|\overset{\cdot }{q},\hat{T},\theta )}}\overset{\text{paired}}{\overset{︷}{\prod_{\hat{q,r}\in S}p(D_{q,r}|\hat{q,r},\hat{T},\theta )}},$$

where $\hat{T}$ is a phylogenetic tree. Felsenstein’s pruning algorithm (Felsenstein 1981) was used to calculate both the unpaired site likelihoods, $p(D_{q}|\overset{\cdot }{q},\hat{T},\theta )$, and the paired site likelihoods, $p(D_{q,r}|\hat{q,r},\hat{T},\theta )$. Paired sites were modeled using the unconstrained M95 model, whereas unpaired sites were modeled using the GTR+Γ model that is nested within the unconstrained M95 model.

### Prior over RNA Secondary Structures

Equation (8) assumes that the secondary structure S is known a priori, either through experimental or computational methods of structure prediction. However, it also possible to treat the secondary structure as unknown, by placing a prior probability distribution, p(S), over secondary structures and marginalizing S.

One way of introducing a prior over secondary structures is by using a Stochastic Context Free Grammar (SCFG). A SCFG is probabilistic extension of a context-free grammar (CFG). A CFG is a type of grammar that defines a set of rules for generating all possible strings in a given formal language. A SCFG extends this notion by assigning probabilities to each possible string in the given language. RNA SCFGs are SCFGs that give probability distributions over strings of base-paired and unpaired nucleotides representing RNA secondary structures (Anderson 2014).

#### The KH99 Grammar

We chose the KH99 SCFG (Knudsen and Hein 1999) as a prior over secondary structures. The rules and associated probabilities for this SCFG are given as follows:

(9) $$G_{\text{KH}99}=\begin{array}{ccccccc} S & \to & • & \text{or} & \mathrm{LS} & \text{or} & \left(F\right)\\ & & 0.118 & & 0.869 & & 0.014\\ L & \to & • & \text{or} & \left(F\right) & & \\ & & 0.895 & & 0.105 & & \\ F & \to & \left(F\right) & \text{or} & \mathrm{LS} & & \\ & & 0.788 & & 0.212. & & \end{array}$$

Note that *S* is the start symbol.

The KH99 assigns probabilities to all strings of a specified length that can be written in dot-bracket notation, with at least two unpaired nucleotides separating every base-pair.

#### Structure-Integrated Likelihood

Using Bayes’ rule, the probability of a secondary structure, S, conditional on the data, D, and phylogenetic parameters, θ, is given by:

(10) $$p(S|D,\theta )=\frac{p(D|S,\theta )p(S)}{p_{S}(D|\theta )}=\frac{p(D|S,\theta )p(S)}{\sum_{S}p(D|S,\theta )p(S)}.$$

We take particular note of the *structure-integrated likelihood* term in the denominator of (10):

(11) $$p_{S}(D|\theta )=\sum_{S}p(D|S,\theta )p(S).$$

This term requires summing over all possible secondary structures and is not a constant that can be ignored due it is dependence on θ. This number grows exponentially with the length of the alignment *L*. Fortunately, there exists an $O(L^{3})$ polynomial-time algorithm, the inside algorithm (Lari and Young 1991), for summing the probabilities of all derivations of an SCFG (all valid secondary structures in the case of RNA SCFG). By analogy to the forward algorithm for HMMs, the inside algorithm allows the structure-integrated likelihood, $p_{S}(D|\theta )$ (the analogue of the forward likelihood for HMMs), to be efficiently computed. The structure-integrated likelihood is given by element *I*(*S*, 1, *L*) of the inside probability matrix, where *S* is the start symbol of the KH99 grammar.

Likewise, by analogy to the backward algorithm for HMMs, there exists an “outside algorithm,” which together with the inside probabilities allows the posterior marginals of the hidden variables to be computed (in the case of an RNA SCFG, these are the positional emission probabilities of base-pair and unpaired terminal symbols—see supplementary section 1.5, Supplementary Material online).

#### Parallelization of the Inside and Outside Algorithms

Supplementary algorithm’s S1 and S2 in Supplementary Material online provide pseudocode for iterative implementations of the inside and outside algorithms for SCFGs in double emission normal form, respectively.

**Fig. 7.**
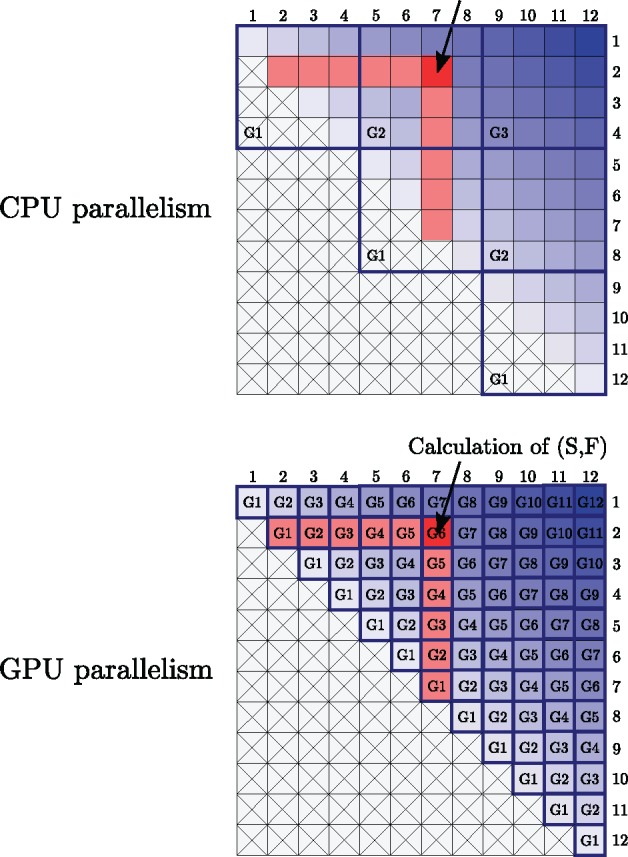
Illustrations of the inside algorithm showing CPU and GPU parallelism schemes. The light to dark blue gradient starting at the central diagonal and finishing in the top right-hand corner indicates the order in which each diagonal is computed. The light red elements indicate the data dependencies required to compute the single bright red entry of the inside matrix. The lower half of each matrix with each cell crossed out is not computed and can be ignored. Note that the top-right element corresponds to the structure-integrated likelihood term and is therefore always the last element to be calculated, as it depends on all other elements having been computed first.

Figure 7 illustrates the calculation of the inside probability matrix, showing the order in which elements are computed and the data dependencies required to compute a particular element. Using these patterns, Sükösd et al. (2011) developed a strategy for CPU parallelism, whereby blocks of elements running diagonally along the inside matrix can be computed in parallel, as they do not have data dependencies. We implemented a similar scheme for the CUDA GPU architecture, whereby instead of blocks, each element along a diagonal is computed in parallel. This can be done because each element along a diagonal is independent of all other elements on the same diagonal. For large alignments (*L *>* *1,000), this implies thousands of computational threads executing the same set of instructions in parallel, but on different data (different elements of a particular diagonal), this is known as SIMD (Single Instruction Multiple Data) parallelism and is the regime of parallelism for which GPU architectures are tailored. As far as we are aware, this is the first GPU implementation of the inside and outside algorithms.

### Paired Site Likelihoods

Because the inside and outside algorithms consider every possible base-pairing they require a matrix **B** of *paired site likelihoods*. Each element ***B***_*qr*_ of **B** corresponds to a paired site likelihood $p(D_{q,r}|\hat{q,r},\hat{T},\theta )$ for a pair of sites, *q* and *r*, in the alignment D, which can be calculated using Felsenstein’s peeling algorithm. Since the diagonal of **B** is ignored and ***B***_*qr*_ = ***B***_*rq*_ (i.e., **B** is symmetric), $\left(\begin{array}{c} L\\ 2 \end{array}\right)$ paired site likelihoods need to be calculated. Although the number of computational steps is only $O(L^{2})$ in the alignment length *L*, compared with $O(L^{3})$ for the inside and outside algorithms, the amount of time per computational step for computing the paired site likelihoods is substantially higher due the use of Felsenstein’s algorithm. To ameliorate this bottleneck, we use the partial site caching strategy of Pond and Muse (2004) to reduce the number of likelihood calculations required and developed a CUDA GPU implementation.

Note that the inside and outside algorithms also require a vector, **S**, of length of *L* single site likelihoods, where each element corresponds to $p(D_{q}|\overset{\cdot }{q},\hat{T},\theta )$. However, this is fast to compute compared with the matrix **B**.

### Sampling Secondary Structure Configurations

The inside probability matrix can be used to sample secondary structure configurations from the distribution:

(12) $$\tilde{S}∼p(S|D,\theta ).$$

Sampling terminal strings (secondary structures in our case) using an SCFG is analogous to sampling hidden state sequences using the forward-filtering backward-sampling algorithm for HMMs (Frühwirth-Schnatter 1994). An algorithmic description for sampling secondary structures from an RNA SCFG is given in supplementary methods section 1.4, Supplementary Material online.

### Bayesian Posterior Inference

The posterior distribution of the continuous-parameters, θ, conditional on the data D and a secondary structure S can be sampled using the Metropolis–Hastings algorithm and the relationship given by Bayes’ formula:

(13) p(θ|D,S)∝p(D|S,θ)p(θ),

where the likelihood term, p(D|S,θ), is given by (8) and p(θ) is the prior.

We can also treat the secondary structure as unknown and assume a RNA SCFG prior, *p*(*S*), over secondary structures. This can be achieved by using the structure-integrated likelihood, $p_{S}(D|\theta )$, when inferring θ:

(14) $$p(\theta |D)\propto p_{S}(D|\theta )p(\theta ).$$

However, note that the structure-integrated likelihood term is computed every time a new set of parameters is proposed. As mentioned previously, this requires computing a matrix **B** of paired site likelihoods (requiring $O(L^{2})$ computational steps) and calculating the final structure-integrated likelihood term using the inside algorithm (requiring $O(L^{3})$ computational steps). Therefore, gathering enough samples to ensure an adequate sample size will be relatively slow. However, given that we can sample the conditional distribution, p(S|D,θ), using the sampling procedure outlined in “Sampling Secondary Structure Configurations” section, this leads to a potentially more efficient Metropolis-within-Gibbs approach. This approach works by alternatively sampling from the full conditional distribution:

(15) $$S^{\left(k\right)}∼p(S|D,\theta^{\left(k\right)})$$

using the sampling procedure outlined in “Sampling Secondary Structure Configurations” section and

(16) $$\theta^{\left(k+1\right)}∼p(\theta |D,S^{\left(k\right)})$$

using the Metropolis–Hastings algorithm. Although the Gibbs sampling step (15) still requires computing a matrix **B** of paired site likelihoods and running the inside algorithm, the Metropolis–Hastings step (16) only requires O(L) operations and can be repeated for multiple iterations following the Gibbs sampling step. In our implementation, we repeat the Metropolis–Hastings step 50 times following the Gibbs sampling step. Together these give a Markov Chain Monte Carlo algorithm whose stationary distribution, p(S,θ|D), and associated marginals, p(S|D) and p(θ|D), are the distributions of interest.

### Maximum Likelihood Inference

The COBYLA optimization algorithm (Powell 1994) in the NLOpt library (Johnson 2014) was used to find the maximum likelihood (ML) parameters via the structure-integrated likelihood (11). Note that when doing so the priors over the continuous parameters were either ignored and estimated using ML, or the priors were used and the parameters were fully marginalized (as specified in the *Priors* section).

### Likelihood Ratio Tests

To test whether the unconstrained model (GU/GT ≥1) was favored over a GU/GT neutral model (GU/GT: = 1) for a particular data set, likelihood ratio tests (LRTs) were performed (table 1 in Results section).

Unfortunately, these LRTs are not entirely valid, because GU/GT ≥1 represents a boundary condition (Self and Liang 1987). The use of a standard LRT with such a boundary condition reduces the probability of rejecting the null hypothesis (GU/GT ≥1). However, we still report the results of these tests because they remain useful in the case of rejection of the null-hypothesis (the test is conservative).

To address this, we performed a bootstrap likelihood ratio test for several data sets. Due to computational limitations, this was only done for 9 out of the 15 recombination-analyzed data sets in table 1. We followed the bootstrapping procedure detailed in Tekle et al. (2016), which was briefly as follows for a given data set:

1. Obtain separate maximum likelihood estimates of the GU/GT neutral (null) model and the unconstrained (alternative) model for the real data set. Calculate the log-likelihood difference.
2. Simulate 20 new data sets of the same size length, and pattern of gaps using the maximum likelihood parameters estimated under the null model.
3. For each simulated data set, re-estimate the maximum likelihood parameters under the GU/GT neutral (null) model and the unconstrained (alternative) model. Calculate the log-likelihood difference for each of the 20 simulated data sets.
4. Obtain an estimate of *P*-value by calculating the position of the log-likelihood difference for the real data set compared with the log-likelihood differences for the simulated data sets.

In addition, due to computational-time constraints, we were also limited to 20 bootstrap simulation in each instance. This implies that a significance threshold of P≈0.05 was used when doing so.

### Data Sets

#### Data Set Construction

Three classes of data sets were analyzed. The first class of data sets consisted of noncoding RNA alignments obtained from version 14.1 of the RFAM database (Burge et al. 2013). We downloaded all 99 RFAM data sets that had an associated consensus secondary structure based on an experimental RNA structure determination. These consensus secondary structures were only used for benchmarks of secondary structure prediction and were not conditioned on during the inference of coevolution rate parameters. RFAM data sets are denoted with “RF” prefix in their name.

The second and third classes of data sets analyzed, consisted of the complete genomes of single-stranded RNA and single-stranded DNA viruses, respectively, obtained from the NCBI nucleotide database (Acland et al. 2014) and aligned using MUSCLE (Edgar 2004). Typically, these data sets did not have associated consensus secondary structures.

A summary of each data set is listed in supplementary table S1, Supplementary Material online. All data sets (alignment and inferred phylogenetic trees) used in this study are available for download from our GitHubrepository.

#### Phylogenetic Inference

Phylogenetic trees were estimated using FastTree (Price et al. 2010) under a GTR+CAT model.

#### Recombination Analysis

About 15 data sets were analyzed for evidence of recombination (those in table 1 and fig. 4) using RDP version 4.97 (Martin et al. 2015) with default settings. Regions of sequences that were detected as recombinant were separated out using RDP’s “Save distributed alignment” option. This option generates expanded alignments with the same sequence content, while reducing the impact of recombination.

Failing to account for recombination can invalidate the assumption of a single phylogenetic tree representing the data. This may impact downstream analyses, particularly in the context of base-pairing coevolution, where rate estimates are based on pairs of nucleotides sites that may not share the same tree. Failing to account for recombination may bias parameter estimates.

It should be noted that when using RDP’s default options, there are likely to be some false positive recombination events. This is not completely undesirable because using these more relaxed settings will capture most of the true positive recombination events that are likely to bias our estimates. Unnecessarily, accounting for false positive recombination events is not expected to bias our estimates.

### Site Permutations

To test whether secondary structure dependencies present in real data sets influence model fit, each alignment was taken and its sites randomly permuted. Two such nucleotide column permuted data sets (*p*_1_ and *p*_2_) were generated for each real data set. ML estimation using the structure-integrated likelihood was used to fit the parameters of each permuted data set under the unconstrained model and the secondary structure information entropy was calculated (see supplementary section 1.7, Supplementary Material online, for a description of how this was calculated). 

## Software Availability

Julia code (compatible with Windows and Linux) is available at: https://github.com/michaelgoldendev/MESSI.

## Supplementary Material

msz243_Supplementary_Data
