# Supplementary figures and images for: Evolutionary Analyses of Base-Pairing Interactions in DNA and RNA Secondary Structures
Source: Mol Biol Evol. 2019 Oct 30;37(2):576–92. doi: 10.1093/molbev/msz243 (PMC6993869; doi:10.1093/molbev/msz243)

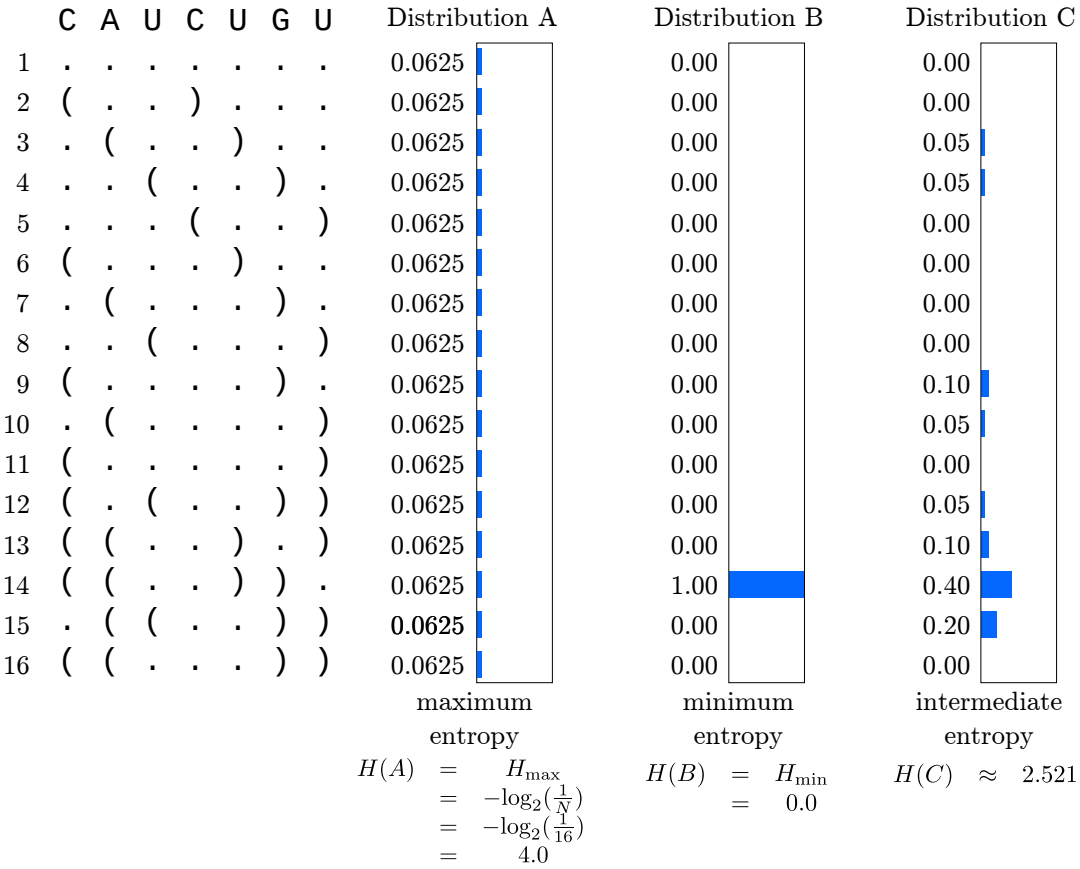

Supplement: msz243_Supplementary_Data [file msz243_supplementary_data.zip › msz243-suppl_data/entropy.pdf]

A

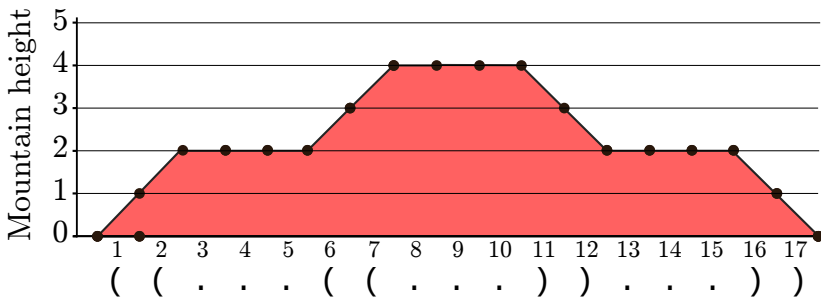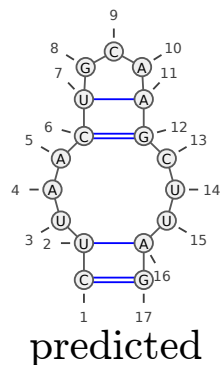

B

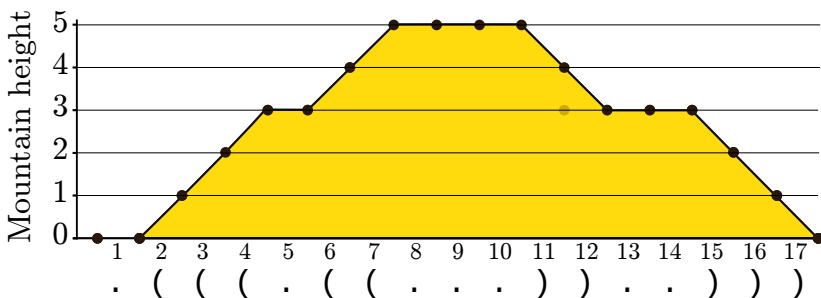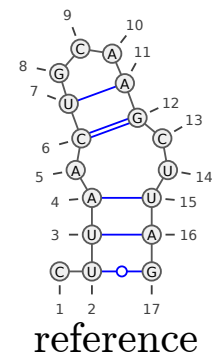

=

C

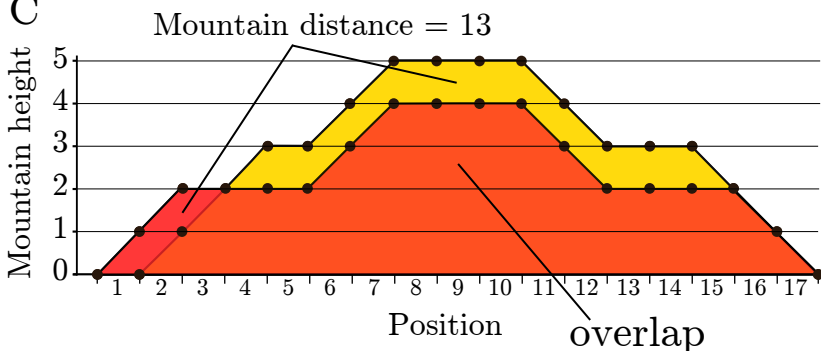

Mountain diameter = 72

Normalised mountain distance =  $13 / 72 = 0.181$

Mountain similarity =  $1 - 0.181 = 0.819$  or 81.9%

Supplement: msz243_Supplementary_Data [file msz243_supplementary_data.zip › msz243-suppl_data/mountain_metric.pdf]

# Outside algorithm timings

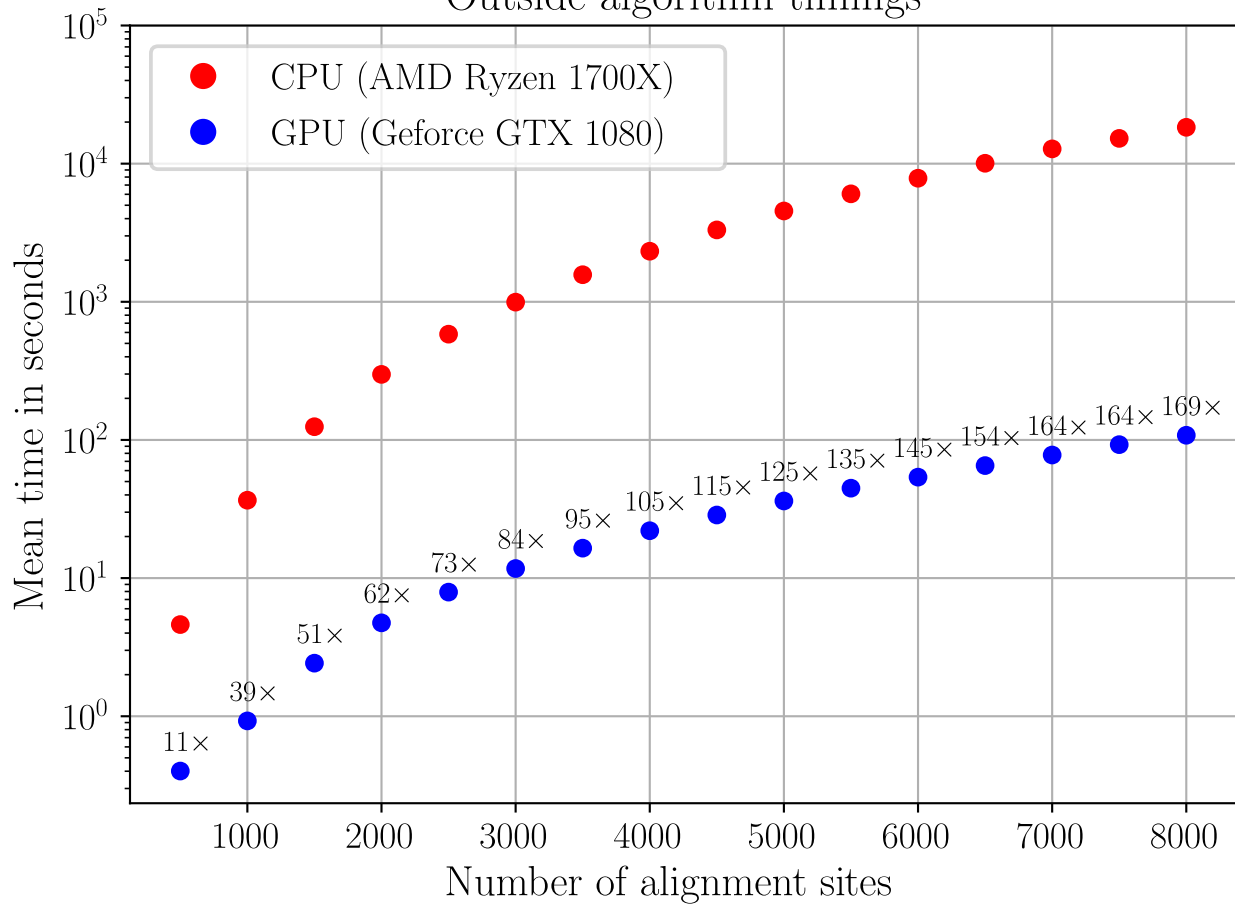

Supplement: msz243_Supplementary_Data [file msz243_supplementary_data.zip › msz243-suppl_data/outside_algorithm_timings.pdf]

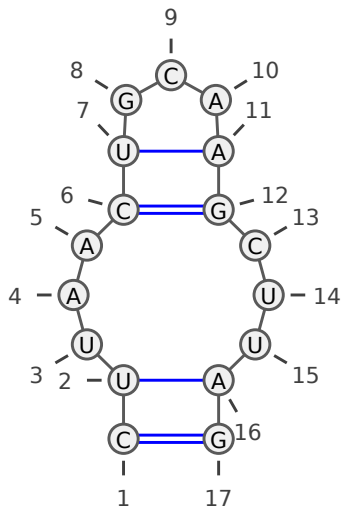

((...((...))...))  
CUUAACUGCAAGCUUAG

predicted

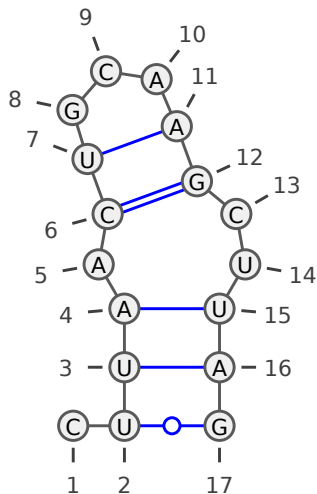

.(((.((...))...))  
CUUAACUGCAAGCUUAG

reference

Supplement: msz243_Supplementary_Data [file msz243_supplementary_data.zip › msz243-suppl_data/predicted_experimental.pdf]
